# Augmented Reality Marketing: A Systematic Literature Review and an Agenda for Future Inquiry

**DOI:** 10.3389/fpsyg.2022.925963

**Published:** 2022-06-16

**Authors:** Zhao Du, Jun Liu, Tianjiao Wang

**Affiliations:** Business School of Sport, Beijing Sport University, Beijing, China

**Keywords:** augmented reality, marketing, retailing, tourism, advertising, technology, brand, tourist destination

## Abstract

Augmented reality (AR) is a potentially disruptive technology that enriches the consumer experience and transforms marketing. With the surging popularity of AR in marketing practice, academic efforts to investigate its effects on consumer experience, response, and behavior have increased significantly. To obtain an integrated and comprehensive view of the front-line in AR marketing research and identify the gaps for future research, we analyze the existing AR marketing literature through a systematic literature review. Using 99 journal articles selected from the Web of Science core collections, this research sheds light on the general characteristics such as publication year, publication outlet, research design, and research method. Moreover, this research also gains insight into the AR marketing relevant factors such as application area, application context, AR type, and theoretical lenses. The findings of the analyses reveal the state-of-the-art of scholarly publications on AR marketing research. First, the number of journal articles on AR marketing increased rapidly in the past few years, and the journals that published articles on AR marketing cover a wide range of disciplines. Second, the empirical studies in most literature adopted the quantitative research design and used survey or experiment methods. Third, the studies in more than half of the journal articles used mobile AR applications in various online contexts. Fourth, the Technology Acceptance Model (TAM) and the Stimulus-Organism-Response (S-O-R) framework are the two most widely used theoretical lenses used in the literature. After that, the major application areas of AR in marketing are retail, tourism, and advertising. To identify the focal themes discussed in the three application areas, this research summarizes the studies by the outcome variables. Specifically, the outcome variables have five categories: technology-related, product-related, brand-related, tourist destination-related, and advertisement-related. Finally, this research proposes the agenda for future academic efforts in AR marketing.

## Introduction

Augmented reality (AR) is an emerging cutting-edge technology in marketing, It enhances the visual, auditory, tactile, and olfactory perception of users by augmenting or superimposing digital content such as text, geolocation information, graphics, audios, and videos onto a live view of the physical objects and environments in real-time (Carmigniani et al., 2011; Fan et al., 2020; Sung, 2021). AR establishes a closer relationship between users’ physical space and virtual objects. Therefore, the user experience with AR is more immersive, more vivid, more interactive, and more realistic (Cipresso et al., 2018). With the popularity of mobile devices and the availability of high-speed wireless networks, an increasing number of web-based AR applications and mobile AR apps have emerged to create novel, immersive, enjoyable, informative, and valuable user experiences. Accordingly, AR is becoming a disruptive technology that will transform marketing in the coming years (Tan et al., 2022). An industry report released by PwC claimed that AR brought net economic benefits of $33 billion in 2019. Furthermore, the benefits will reach $338.1 billion by 2025 and $1.0924 trillion by 2030 (PwC, 2019).

The surging popularity of AR in marketing practice has attracted more and more academic efforts to investigate its effects on consumer experience, response, and behavior (Rauschnabel et al., 2022). This growing interest in AR marketing calls for a synthesis of the existing literature to offer guidance for future research. However, as scholarly investigations on AR marketing are still in the infant stage, the extant literature on AR marketing is fragmented. In this regard, we analyze AR marketing literature through a systematic literature review to obtain an integrated and comprehensive view of the state-of-the-art of AR marketing research and identify the gaps for future research. Specifically, this research sheds light on the generic characteristics of the literature, such as publication year, publication outlet, research design, and research method. In addition, this research also gains insight into the factors specific to AR marketing, such as application area, application context, AR type, and theoretical lenses. We also identify the focal themes in each application area according to the outcome variables to illustrate the current status of scholarly investigation. Moreover, we propose the agenda for future research.

This systematic review differs from existing literature reviews in four ways. First, this review conducts an extensive examination of AR marketing literature. We initially identified 442 journal articles from 200 journals for manual evaluation. These journal articles cover a publication period from 2000 to 2021. After assessing the details following the guidelines of a systematic review methodology, we have 99 journal articles in the final set for analysis. Second, this study adopts a systematic review approach, thus allowing better synthesis and integration. It can help AR marketing researchers better understand existing findings and identify potential topics for future research. Third, this research gains insight into the factors specific to AR marketing, such as application areas, application context, AR type, and theoretical lenses, and summarizes the literature in terms of these factors. Finally, this research identifies and categorizes the AR marketing literature by its application areas, which offers a new perspective to gain insight into the state-of-the-art of AR marketing research.

The remainder of this paper is organized as follows. First, we present the concept of AR and discuss its application in marketing. First, we present the concept of AR and discuss its applications in marketing. Second, we explain the methodology used in the literature search and select the journal articles reviewed in this study. Third, we summarize the journal articles for final analysis in terms of the general factors (e.g., publication year, publication journal, research design, and research method) and the AR relevant factors (e.g., application area, AR type, application context, and theoretical lenses). Fourth, we analyze the focal themes in the three application areas of retail, tourism, and advertising. Lastly, we present the contributions and concluding remarks, future research agenda, and limitations of this research.

## Augmented Reality and Augmented Reality Marketing

### Augmented Reality

Augmented reality originated from Morton Heilig’s bold and innovative idea that cinema needed to draw viewers into the onscreen activities by effectively taking in all senses (Carmigniani et al., 2011). Although we can track the history of AR back to the 1950s, the way of AR from laboratories to the industry has taken more than half a century. The exposure of AR to a mass audience has not realized until the explosive popularity of Pokémon GO in 2016, which provided both the social and fashionable acceptance for the success of AR in the market (Rauschnabel et al., 2017).

Augmented reality is built on computer vision and object recognition technologies. It enhances consumer experiences by augmenting or superimposing digital content (e.g., text, geolocation information, graphics, audio, and videos onto) a live view of the physical objects and environments (e.g., consumers’ faces, bodies, and surroundings) in real-time (Sung, 2021). The discussions of AR and Virtual Reality (VR) usually connect closely. Compared with traditional media, AR and VR aim to provide users with enriched, interactive, and immersive media experiences (Yim et al., 2017). While VR creates a fully computer-generated virtual environment, AR enriches the real environment by integrating context-aware digital information (Huang and Liao, 2015; Yim et al., 2017).

A typical AR system consists of three components: a geospatial datum for the virtual object, a surface to project virtual elements, and an image processing unit (Carmigniani et al., 2011). Early AR systems have limited applications in business practices. They need to be built on dedicated devices such as smart glasses (e.g., HoloLens Magic and Google Glass) (Poushneh, 2018), somatosensory devices (e.g., Kinect) (Huang and Liao, 2017; Huang, 2021), or fixed devices (e.g., PC and its connected webcam) and smart mirror (Rese et al., 2017; Baek et al., 2018). Recently, with the prevalence of personal mobile devices (e.g., smartphones and tablets) and the availability of high-speed wireless networks, the application of AR has proliferated in a variety of fields such as education (Wu et al., 2013), manufacturing (Nee et al., 2012), healthcare (Ferrari et al., 2019), and marketing (Tan et al., 2022).

### Augmented Reality Marketing

Augmented reality marketing refers to the application of AR in marketing to enhance consumers’ experiences, increase their satisfaction, shape their behavior, and boost companies’ revenues (Huang and Liao, 2015; Javornik, 2016; Poushneh and Vasquez-Parraga, 2017; Bell et al., 2018). The novel and attractive media of presentation and interaction enabled by AR play a crucial role in achieving the desired effects. Specifically, AR integrates digital information or objects into consumers’ perceptions of the physical objects and environments, thus providing consumers with rich information about products or services and allowing them to experience products and services easily. Specifically, AR not only improves online experiences and engagement but creates novel and fantastic on-site experiences (Javornik, 2016; Yuan et al., 2021).

First, AR engages consumers in online settings by providing real-time direct product/service experiences in various aspects of marketing (Chung et al., 2018). Specifically, it overcomes the limitations of online shopping by allowing prospects to try on products, such as makeup (Smink et al., 2019; Hsu et al., 2021; Javornik et al., 2021), eyewear (Pantano et al., 2017; Yim et al., 2017; Yim and Park, 2019), clothing (Huang and Liu, 2014; Huang and Liao, 2017; Plotkina and Saurel, 2019), shoes (Hilken et al., 2018; Plotkina et al., 2021), and furniture (Rauschnabel et al., 2019; Kowalczuk et al., 2021; Qin et al., 2021b) virtually without having to interact physically with them. Major online retailing platforms, such as Amazon (McLean and Wilson, 2019), JingDong (Fan et al., 2020), Alibaba (Fan et al., 2020), and eBay (Banerjee and Longstreet, 2016), as well as leading brands, such as Tiffany & Co. (Whang et al., 2021), L’Oréal (Hilken et al., 2017), Sephora (Smink et al., 2019), Nike (Hilken et al., 2018), Converse (Whang et al., 2021), Zara (Yuan et al., 2021), IKEA (McLean and Wilson, 2019; Qin et al., 2021b), Mini (Carmigniani et al., 2011), and Lego (Hinsch et al., 2020), have devoted lots of efforts to introduce various forms of AR. They strive to enhance consumers’ vicarious experience of physical products in online settings and make it more immersive, interactive, informative, enjoyable, and satisfactory (Yim et al., 2017). Furthermore, AR advertising has significant advantages over traditional advertising. AR empowered advertisements are more informative, novel, entertaining, and complex, which leads to positive consumer responses and helps advertising campaigns stand out (Feng and Xie, 2018; Yang et al., 2020; Sung, 2021).

Second, AR offers a novel and fantastic on-site experience (Barhorst et al., 2021). The application of AR creates augmented stores (Bonetti et al., 2019), restaurants (Heller et al., 2019a; Batat, 2021), museums (tom Dieck et al., 2016; He et al., 2018; Zhuang et al., 2021), and art galleries (tom Dieck et al., 2018b; Tussyadiah et al., 2018). Retail giants, such as Lowes (Chalimov, 2021) and Machine-A (Chitrakorn, 2021), engage consumers and offer interaction by incorporating AR-supported features into their mobile apps and serving consumers in innovative ways. Furthermore, both established and novel brands, such as Kate Spade, Charlotte Tilbury, Timberland, Lily, Philip, Lego, and Toys-R-Us, offer consumers a plethora of interactive experiences. The interactive experiences include learning more about products, creating unique and customizable products, and virtually trying on products by installing in-store AR displays or adding AR empowered features to the brand’s mobile apps (Chalimov, 2021). AR augmented stores can produce extra brand value, simplify consumers’ decision-making process, stimulate brand engagement, and lead to stronger consumer purchase desire (Bonetti et al., 2019; Cuomo et al., 2020). AR-empowered restaurant services affect consumers’ perceptions of restaurant experiences (Batat, 2021) and promote the choice of high-value products (Heller et al., 2019a). Moreover, augmented reality applications, especially those built upon wearable devices, affect tourists’ destination visit intention (Chung et al., 2015). They can also help tourists feel more enjoyable (Tussyadiah et al., 2018), enhance their experiences with tourist destinations (tom Dieck et al., 2018a; Jiang et al., 2019), and increase their willingness to pay more (He et al., 2018).

## Methodology

This research adopts the systematic literature review approach to avoid the well-known limitations of literature selection in narrative reviews and expert reviews (Tranfield et al., 2003; Kitchenham et al., 2009) and synthesize the existing research findings in a transparent and reproducible way (Snyder, 2019). Following the guidelines for the systematic review approach (Webster and Watson, 2002; Denyer and Tranfield, 2009; Paul and Criado, 2020), we conducted a review of AR marketing to identify relevant themes for this field. The guidelines suggest five steps for producing a systematic review that is both reproducible and transparent (Snyder, 2019). The five steps include question formulation, study location, study selection and evaluation, analysis and synthesis, and results reporting and using (Denyer and Tranfield, 2009).

### Question Formulation

Question formulation is crucial for a well-conducted systematic review. To obtain a deeper understanding of the AR marketing literature, we conduct a pilot search in the first stage. Based on the findings of the pilot search, we establish the research scope, formulate research questions, and clarify the inclusion and exclusion criteria. The pilot search leads us to the central questions of this research: what are the roles of AR in marketing? and how does AR contribute to marketing? Specifically, we propose four research questions: (RQ1) How is AR marketing defined in the literature? (RQ2) What are the characteristics of the AR marketing literature? (RQ3) What are the major application areas investigated by the AR marketing literature? and (RQ4) What are the focal themes examined in each application area?

### Study Location

We perform the first search with the term “augmented reality” in the Web of Science (WOS) core collections, which return 9,145 journal articles on the subject of AR. However, most papers come from WOS categories such as Computer Science, Engineering, Medical, Education, and other fields not related to marketing. Therefore, we perform a second search in the WOS core collections using the following query applied to the title, abstract, and keywords: [“augmented reality” AND (marketing OR consumer OR customer)]. As this research focuses on the most relevant studies of AR marketing, we keep the papers from the five WOS categories, such as Business, Management, Hospitality Leisure Sport Tourism, Computer Science Information Systems, and Computer Science Interdisciplinary Applications (Alves et al., 2016). Furthermore, we limit the publication year to “1990–2021,” language to English, and document type to Article. From the query, we get 341 journal articles.

To ensure that no major AR marketing articles are ignored in the analysis, we use a “snowball” technique. Specifically, we review citations from the key studies in the 341 journal articles retrieved in the previous search and identify more keywords related to AR marketing. After that, we perform the third search using the following query applied to the title, abstract, and keywords: [“augmented reality” AND (marketing OR consumer OR customer OR retail* OR advertis* OR brand* OR touris*)]. There are multiple possible terms under the root word. Hence, some of the words in the query are followed by a wildcard. Meanwhile, we also limit the five WOS categories in the previous search, the publication year to “1990–2021,” language to “English,” and document type to “Article.” From the query, we obtain 442 journal articles.

### Study Selection and Evaluation

For the 442 journal articles, we conduct manual screening of the titles, keywords, abstracts, and text under the following three inclusion and exclusion criteria:

(1) The study should focus on AR. Thus, we not only exclude the journal articles that discuss VR, XR, AI, and emerging innovative techniques but their combinations with AR.
(2) We focus on AR applications in the marketing context. Therefore, we exclude the articles that discuss the technical details of AR and AR-empowered systems and the application of AR in the non-marketing context.
(3) We aim to shed light on the findings of empirical studies. Hence, we exclude the conceptual papers and review papers.

**FIGURE 1:**
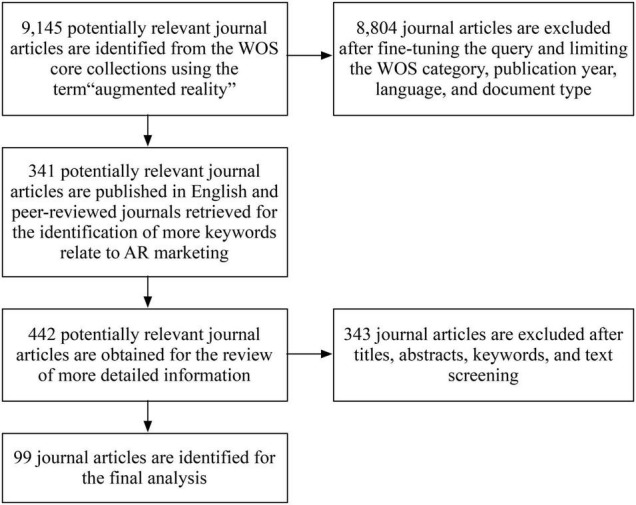
The process of selecting the journal articles for final analysis.

After removing 343 journal articles, there are 99 journal articles included in the final set for further content analysis. During the screening process, two authors first identify potentially relevant articles independently. Then, we discuss the conflicts to obtain the journal articles for final analysis so that the agreement (Cohen’s Kappa coefficient) is larger than 0.85. Figure 1 presents the process used to select the journal articles for final analysis.

### Analysis and Synthesis

The authors manually develop a data extraction process to report the main characteristics of the journal articles, such as publication year, publication journal, application areas, application context, AR type, research design, research method, and theoretical lenses. Then, two authors independently code the selected journal articles in themes, which offer us a more comprehensive understanding.

## Descriptive Analysis

In this section, we summarize the 99 journal articles on AR marketing by examining the general characteristics such as publication year, publication outlet, research design, and research method, as well as the AR relevant characteristics such as application area, application context, AR type, and theoretical lenses.

### Publication Year

**FIGURE 2:**
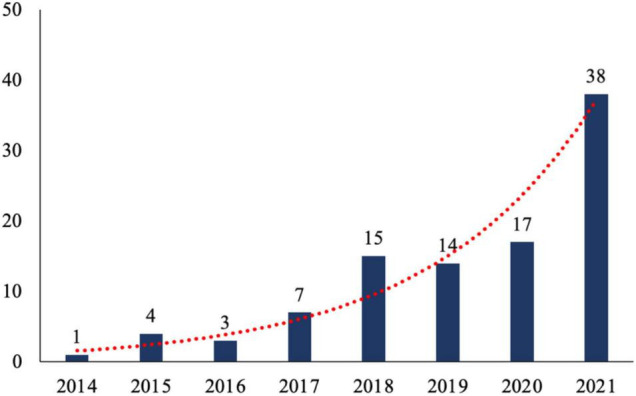
Publication year.

As presented in Figure 2, the number of journal articles published on AR marketing keeps increasing from 2014 to 2021. Specifically, the first article is published in 2014 (Huang and Liu, 2014). Then, the number of journal articles increases rapidly from 2015 to 2018. After that, the number of articles goes up slowly from 2019 to 2020 and surges in 2021. In particular, the number of journal articles published in 2021 is more than two times the number of journal articles published in 2020. The fast growth of publications is consistent with the proliferation of AR applications in marketing practices. Specifically, the number of global mobile AR users reached 200 million in 2015. It will grow to 1.1 billion in 2022 and 1.7 billion in 2024 (Alsop, 2021).

### Publication Journal

The 99 articles are published in 43 journals of which 25 journals only have one article and nine journals just have two articles. Table 1 presents the nine journals that have three articles or above. Among them, *Journal of Retailing and Consumer Services* has 22 articles, which ranks first in all journals. The number of journal articles published in this journal accounts for more than one-fifth of all publications. *Journal of Business Research* has nine articles, which ranks second in all journals. *Computers in Human Behavior* and *Technological Forecasting and Social Change* tie for the third place with four articles each.

**TABLE 1:** Publication journal.

Journal	# Articles
*Journal of Retailing and Consumer Services*	22
*Journal of Business Research*	9
*Computers in Human Behavior*	4
*Technological Forecasting and Social Change*	4
*Current Issues in Tourism*	4
*Journal of Hospitality and Tourism Technology*	4
*Internet Research*	3
*Tourism Management*	3
*International Journal of Advertising*	3

**FIGURE 3:**
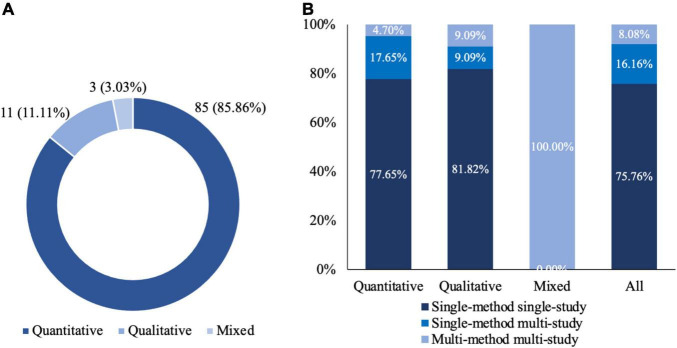
Research design. **(A)** Distribution of qualitative, quantitative, and mixed studies. **(B)** Composition of research methodologies.

Figure 3 shows the research design used in the selected AR marketing literature. We first analyze the research design by the broad category, that is, quantitative, qualitative, and mixed research design. The quantitative research design incorporates quantitative methods such as surveys and experiments. The qualitative research design adopts qualitative methods such as interviews and focus groups. Finally, the mixed research design uses both quantitative and qualitative methods (Sreejesh and Mohapatra, 2014). As presented in Figure 3A, the quantitative methodology dominated the field. Specifically, 85.86% of the journal articles adopts the quantitative research design, 11.11% of the journal articles uses the qualitative research design. Only 3.03% of the corpus takes the mixed research design.

Second, a fine-grained examination of the research design by single-method vs. multi-method and single-study vs. multi-study demonstrates that a considerate proportion of the journal articles use the multi-method or multi-study research design. Specifically, the single-method research design or multi-method research design refers to whether there are one or multiple research methods, such as survey, experiment, interview, and focus group, in the research design. Moreover, the single-study research design or multi-study research design refers to whether there are one or multiple studies in the research design. It is noteworthy that a single study can only use one research method. However, a single-method research design may have one or multiple studies. Thus, from the perspectives of single-method vs. multi-method and single-study vs. multi-study, we have three types of research design: single-method single-study, single-method multi-study, and multi-method multi-study. As presented in Figure 3B, most journal articles (75.76%) use the single-method single-study research design. Only about a quarter of the journal articles (16.16% + 8.08% = 24.24%) adopt the single-method multi-study or multi-method multi-study research design. The proportion of the journal articles using the single-method multi-study or multi-method multi-study research design among the journal articles using the quantitative research design (17.65% + 4.71% = 22.35%) is slightly higher than that among the journal articles using the qualitative research design (9.09% + 9.09% = 18.18%).

### Research Method

Table 2 presents the number and the ratio of the journal articles that use different research methods in the selected AR marketing literature. The most popular research methods are the experiment and survey, which are adopted by 43.44 and 39.39% of the journal articles, respectively. The statistics are consistent with the fact that most research in this field is consumer/tourist-oriented. Specifically, the survey studies can be the online survey, the offline survey, and those performed by survey companies. The experiment studies can be the lab experiment, the online experiment, and the field experiment. Among the qualitative methods, the interview is more popular than the focus group. In multi-method studies, the combination of experiment and survey is the most commonly used. Furthermore, scholars often use the combinations of the interview and one of the other three methods.

**TABLE 2:** Research design.

Research design	Method	# Articles	Ratio
Single-method	Survey	43	43.44%
	Experiment	39	39.39%
	Interview	8	8.08%
	Focus group	1	1.01%
Multi-method		8	8.08%

### Application Area

We identify retailing, tourism, and advertising as the three major application areas of AR marketing. Specifically, AR marketing research originates from retail. The studies in the first journal article and most of the early journal articles are conducted in retail. Moreover, the number of journal articles that shed light on AR in retail has increased significantly over the last few years. Tourism is the second application area of AR marketing. However, the increase in the number of journal articles on AR in tourism keeps steady after the first years. Advertising is the latest application area of AR in marketing. The first journal article on AR in advertising is published in 2018. Nevertheless, the number of journal articles on this topic remained limited until 2021.

**FIGURE 4:**
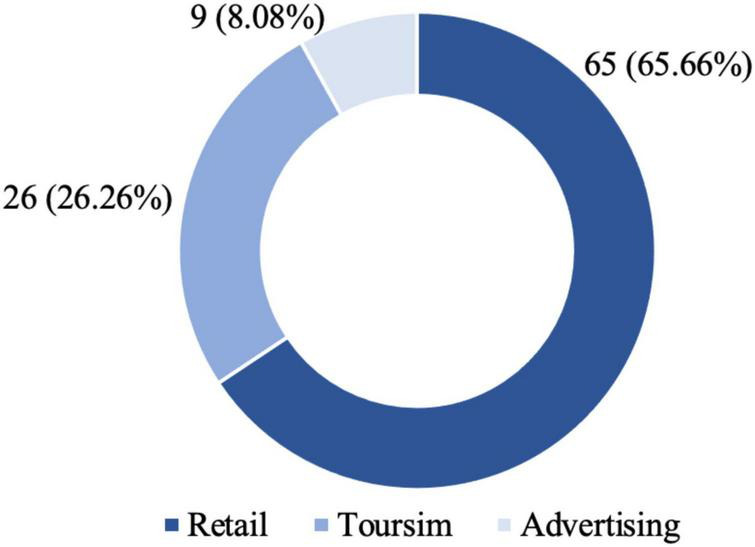
Application area.

Figure 4 presents the number and ratio of the journal articles for the three application areas. First, retail is the earliest application area that attracted the most attention. There are 65 journal articles (65.66%) that shed light on AR in retailing. Second, scholarly works on AR in tourism appears shortly after those on AR in retail. Tourism ranks second in terms of the number of journal articles among the three application areas. There are 26 journal articles (26.26%) that have gained insight into AR in tourism. Finally, advertising is the newest application area that has received the least attention. Specifically, only eight journal articles (8.08%) have examined AR in advertising.

### Application Context

The application context of AR includes both online settings and on-site scenarios. In online settings, AR enriches consumers’/tourists’ experience and improves their satisfaction with online retail, virtual tourism, and online advertising (Chung et al., 2018). In on-site scenarios, AR increases the attractiveness of physical stores, restaurants, museums, and art galleries by offering novel and fantastic experience to consumers/tourists (Barhorst et al., 2021).

**FIGURE 5:**
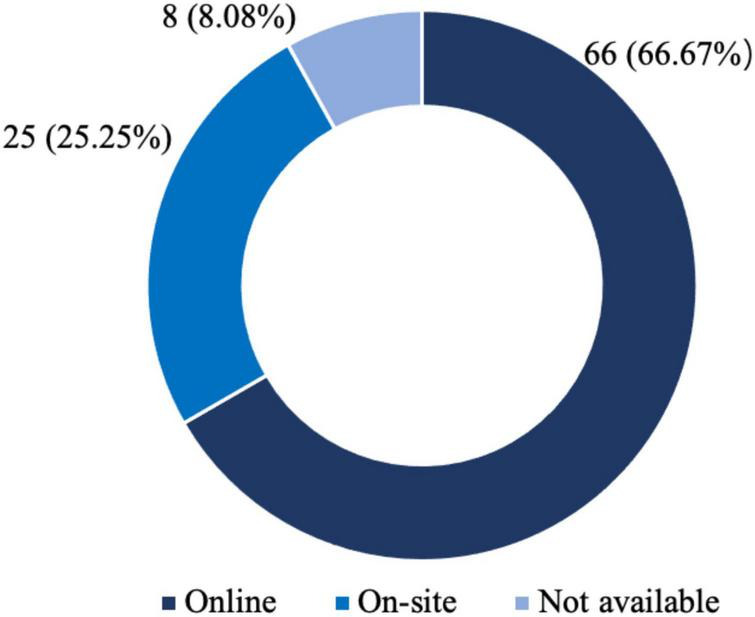
Application context.

Figure 5 presents the distribution of application context in the selected AR marketing literature. While most journal articles investigate the AR application in online settings, a quarter of the journal articles gain insight into AR applications in offline scenarios. First, the studies in 66 articles (66.67%) have focused on the AR application in online settings. The majority of existing studies on the AR application in retail and all prior studies on the AR application in advertising used online settings. Second, the studies in 25 journal articles (25.25%) concentrate on the application of AR in offline scenarios. A considerable proportion of the studies focus on the application of AR in tourism used offline scenarios.

### Augmented Reality Type

The AR applications that consumers interact with are built upon stationary devices (e.g., AR mirrors and PC), mobile devices (e.g., smartphones and tablets), wearable devices (e.g., headsets and smart glasses), and somatosensory devices (e.g., Kinect) (Rauschnabel, 2018). Accordingly, the application of AR in marketing has different types, such as web-based AR, mobile AR, somatosensory device-based AR, wearable AR, and on-site AR.

Early applications of AR in marketing are web-based. In particular, consumers experience the products such as sunglasses, watches, makeup, clothes, shoes, and furniture through web-based AR applications (e.g., virtual try-on) installed on their PCs (Huang and Liu, 2014; Huang and Liao, 2015; Huang and Tseng, 2015). Specifically, they also need to have webcams connected to their PCs. Later on, with the widespread use of mobile devices that have innovative sensors (e.g., smartphones and tablets) and the availability of economic and high-speed mobile internet, mobile AR apps have gained popularity rapidly due to their convenience and low cost. Nowadays, AR is predominantly available in more and more mobile apps (eMarketer, 2020). Meanwhile, AR is also in more sophisticated forms. Specifically, consumers need to experience AR using smart glasses (e.g., HoloLens) (Carrozzi et al., 2019; Heller et al., 2019b) or somatosensory devices (e.g., Kinect and depth sensors) (Huang and Liao, 2017; Huang, 2018; Huang et al., 2019). Furthermore, it is worth noting that AR is prevalent in both online settings and on-site scenarios (Barhorst et al., 2021).

**FIGURE 6:**
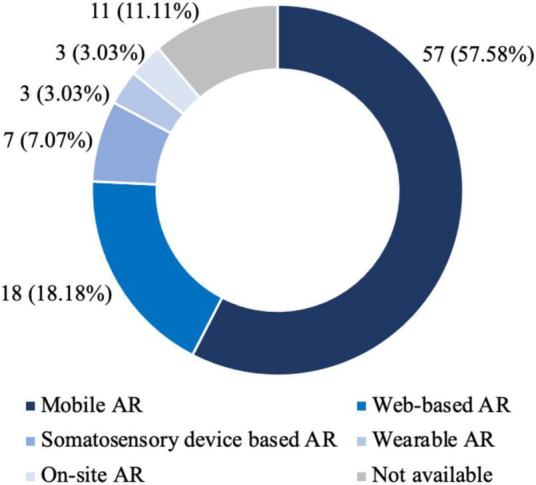
Augmented reality type.

Figure 6 presents the distribution of AR types for the selected AR marketing literature. First, mobile AR is the dominant AR type. The studies in 57 journal articles (57.58%) use mobile AR as the research context. Second, the popularity of web-based AR follows mobile AR. The studies in 18 journal articles (18.18%) use web-based AR. Third, somatosensory device-based AR, wearable AR, and on-site AR are rare AR types. Only several journal articles use these AR types. Finally, no clear AR type is claimed in 11 journal articles. A closer look at the relationship between application areas and AR types shows that mobile AR and web-based AR are prevalent across the three application areas of retail, tourism, and advertising. Specifically, mobile AR is the dominant AR type. However, on-site AR only exist in retailing, wearable AR only exists in tourism, and somatosensory device-based AR has not appeared in advertising.

### Theoretical Lenses

Extant AR marketing literature builds upon a wide range of theory lenses. Table 3 summarizes the nine popular theoretical lenses used in at least three journal articles. Specifically, Technology Acceptance Model (TAM) is the most popular one. Altogether, 15 journal articles use TAM in the studies. Meanwhile, Stimulus-Organism-Response (S-O-R) Framework is also a widely used theoretical framework in AR marketing literature, which has appeared in seven journal articles. Furthermore, Self-referencing Theory, Use and Gratification Theory (UGT), Equity Theory, Flow Theory, Theory of Reasoned Action (TRA), and Unified Theory of Acceptance and the Use of Technology (UTAUT) are also well accepted theoretical perspectives.

**TABLE 3:** Theoretical lenses.

Theory	Description	References
Technology Acceptance Model (TAM)	The theory suggests that individuals’ perception of ease of use and usefulness determines their attitude toward a technological system and behavioral intention of using it	Chung et al., 2015; Huang and Liao, 2015; Pantano et al., 2017; Rese et al., 2017; tom Dieck and Jung, 2018; Jiang et al., 2019; McLean and Wilson, 2019; Plotkina and Saurel, 2019; Cuomo et al., 2020; Park and Yoo, 2020; Castillo and Bigne, 2021; Manchanda and Deb, 2021; Saleem et al., 2021; Srivastava et al., 2021; Zhuang et al., 2021
Stimulus-Organism-Response (S-O-R) Framework	The framework suggests that the various aspects of the environment (i.e., stimulus) evoke consumers’ cognitive and affective states (i.e., organism), and subsequently affect their approach or avoidance behaviors (i.e., response)	Baytar et al., 2020; Daassi and Debbabi, 2021; Han et al., 2021; Hsu et al., 2021; Nikhashemi et al., 2021; Qin et al., 2021b; Whang et al., 2021
Self-Referencing Theory	The theory suggests that self-referencing (i.e., the cognitive process of personally relating to information) heightens memories of advertisement information and enhances product and brand evaluations	Huang and Tseng, 2015; Baek et al., 2018; Huang, 2019; Phua and Kim, 2018
Uses and Gratification Theory (UGT)	The theory suggests that media usefulness is the cognitive gratification that stimulates media usage intentions	Rauschnabel, 2018; Kowalczuk et al., 2021; Nikhashemi et al., 2021; Zhang et al., 2019
Equity Theory	The theory suggests that individuals’ decision-making of using a technology depends on their comparison of the input or benefits (i.e., what they receive) and the outputs or costs (i.e., what they sacrifice)	Poushneh and Vasquez-Parraga, 2017; Poushneh, 2018; Smink et al., 2019
Flow Theory	The theory suggests that individuals can achieve the flow experience by implementing the design aspects that facilitate the optimal states of flow. Moreover, the flow experience will enhance other consumer experience outcomes	Huang and Liao, 2017; Barhorst et al., 2021; Yuan et al., 2021
Theory of Reasoned Action (TRA)	The theory suggests that individuals’ behavior can be predicted by their intentions. Moreover, the intentions are determined by their attitudes toward the behavior	Chung et al., 2018; Lacka, 2020; Park and Yoo, 2020
Unified Theory of Acceptance and the Use of Technology (UTAUT)	The theory suggests that performance expectancy, effort expectancy, social influence, and facilitating conditions are direct determinants of behavioral intention	Paulo et al., 2018; Saprikis et al., 2021; Wu and Lai, 2021

## Thematic Analysis

To have a fine-grained understanding of the AR marketing literature, we summarize the focal themes in the three application areas (e.g., retail, tourism, and advertising) based on the outcome variables. Table 4 presents the outcome variables and their categories. To begin with, the literature on AR in retail examines technology-related, product-related, and brand-related outcome variables. Second, the literature on AR in tourism investigates the technology-related and tourist destination-related outcome variables. Finally, the outcome variables explored in the literature on AR in advertising include advertisement-related, brand-related, and product-related outcome variables.

**TABLE 4:** Outcome variable.

Application area	Category	Outcome variable
Retail	Technology-related	Consumers’ attitude toward, satisfaction with, adoption/use intention of, continued use/reuse intention of, and recommendation intention of AR technology/AR retail application
	Product-related	Consumers’ product attitude, product purchase intention, willingness to pay a price premium, and WOM intention
	Brand-related	Consumers’ brand attitude, perceived brand personality, and brand purchase intention
Tourism	Technology-related	Tourists’ attitude toward, adoption intention of, satisfaction with, and recommendation intention of AR technology/AR tourism application
	Tourist destination-related	Tourists’ knowledge acquisition of, visit intention of, satisfaction with, and memory of tourist destinations; tourists’ choice of products and willingness to pay a price premium in tourist destinations
Advertising	Advertisement-related	Consumers’ attitude toward advertisements
	Brand-related	Consumers’ brand attitude and brand liking
	Product-related	Consumers’ product purchase intention

### Retail

Retail is the earliest application area of AR in marketing. Recently, the recognition and adoption of AR marketing by retail giants, leading on-site retailers, and well-known consumer brands have increased significantly. Both industrial practice and academic research have provided evidence for the potentials of AR to entertain, educate, and engage consumers. Specifically, AR can transform online and on-site experience, inspire brand love, facilitate pre-purchase product fit evaluation, boost product sales, and enhance post-purchase consumption experience (Tan et al., 2022). Extant literature on the application of AR in retail has investigated the effects of AR use and various AR characteristics on a set of technology-related, product-related, and brand-related outcome variables and shed light on the underlying mechanisms of these effects. Some articles focus on a specific category of outcome variables (i.e., technology-related, product-related, or brand-related outcome variables); others examine more than one category of outcome variables.

Table 5 presents the popular AR characteristics examined in the literature on AR in retail. The characteristics include interactivity, augmentation, informativeness, vividness, novelty, and aesthetics. Interactivity is the most widely investigated AR characteristic. It refers to the capability of a technological system to enable users to interact easily, control, manipulate, and be involved with the content. Augmentation, also called augmentation quality, is the most unique characteristic of AR that offers an immersive consumer experience. It describes the extent to which the digital objects are integrated into a person’s real-world environment and the ability to enable users to move the digital objects naturally. Informativeness describes the degree to which the provided information is beneficial for better decision-making of consumers. Vividness refers to the ability of AR to combine the sensory experience of real objects (e.g., that can be seen and touched) with the non-sensory imaginary objects (i.e., those created in an individual’s mind) to create a clear image of a product or experience for consumers. Novelty describes the newness, uniqueness, specificness, and unusualness of the AR-enriched information that are presented to consumers. Aesthetics describes the visual appeal of AR-enriched objects or AR empowered environments.

**TABLE 5:** Augmented reality characteristics examined in the literature on AR in retail.

AR characteristic	Description	References
Interactivity	It refers to the capability of an AR system to enable consumers to interact easily, control, manipulate, and be involved with the content.	Pantano et al., 2017; Yim et al., 2017; McLean and Wilson, 2019; Yim and Park, 2019; Park and Yoo, 2020; Barhorst et al., 2021; Hsu et al., 2021; Kowalczuk et al., 2021; Nikhashemi et al., 2021; Poushneh, 2021; Qin et al., 2021b; Whang et al., 2021
Augmentation	It describes the extent to which the digital objects are integrated into the real-world environment and the ability to enable consumers to move the digital objects naturally	Javornik, 2016; Hilken et al., 2017; Rauschnabel et al., 2019; Song et al., 2019; Fan et al., 2020; Hinsch et al., 2020; Daassi and Debbabi, 2021; Nikhashemi et al., 2021; Poushneh, 2021
Informativeness	It refers to the degree to which the provided information is helpful for consumers’ decision-making	Pantano et al., 2017; Rese et al., 2017; Smink et al., 2019; Chiu et al., 2021; Hsu et al., 2021; Kowalczuk et al., 2021; Qin et al., 2021b; Yuan et al., 2021
Vividness	It describes the ability of AR to create a clear image of a product or experience for consumers by combining the sensory experience of real objects with the non-sensory imaginary objects	Yim et al., 2017; McLean and Wilson, 2019; Barhorst et al., 2021; Nikhashemi et al., 2021; Whang et al., 2021
Novelty	It refers to the newness, uniqueness, specificness, and unusual of the AR enriched information that consumers are presented with	Yim et al., 2017; McLean and Wilson, 2019; Yim and Park, 2019; Barhorst et al., 2021; Nikhashemi et al., 2021; Yuan et al., 2021
Aesthetics	It describes the visual appeal of AR enriched objects or AR empowered environments	Huang and Liu, 2014; Huang and Liao, 2015; Pantano et al., 2017; Yuan et al., 2021

#### Technology-Related Outcome Variables

Technology-related outcome variables examined in the literature on AR in retail include consumers’ attitude toward, satisfaction with, adoption/use intention of, continued use/reuse intention of, and recommendation intention of AR technology/AR retail application (e.g., web-based AR retail application and mobile AR retail app). Among these outcome variables, consumers’ attitude toward and reuse intention of AR technology/AR retail application are the most popular. It is noteworthy that many journal articles examined two or more responses simultaneously. In particular, consumer attitude toward AR technology/AR retail application is frequently investigated together with their adoption/use intention of, recommend intention of, and reuse use intention of AR technology/AR retail application.

Consumers’ attitude toward AR technology/AR retail application refers to their feelings associated with using it (Pantano et al., 2017; Rese et al., 2017; Yim et al., 2017; Plotkina and Saurel, 2019; Yim and Park, 2019; Park and Yoo, 2020; Smink et al., 2020; Daassi and Debbabi, 2021; Qin et al., 2021b). Consumers’ satisfaction with AR technology/AR retail application describes their accumulative feelings when interacting with it repetitively within a period (Poushneh and Vasquez-Parraga, 2017; Chiu et al., 2021). Consumers’ adoption/use intention of AR technology/AR retail application refers to their willingness to adopt/use it (Pantano et al., 2017; Rese et al., 2017; Yim and Park, 2019; Bonnin, 2020; Park and Yoo, 2020; Qin et al., 2021b). Consumers’ continued use/reuse intention of AR technology/AR retail application describes their willingness to use it again in the future (Javornik, 2016; Pantano et al., 2017; Chiu et al., 2021; Daassi and Debbabi, 2021; Hsu et al., 2021; Kowalczuk et al., 2021; Nikhashemi et al., 2021). Consumers’ recommendation intention for AR technology/AR retail application refers to their willingness to share the information about it with friends privately or on social media publicly (Javornik, 2016; Pantano et al., 2017; Park and Yoo, 2020; Smink et al., 2020).

The literature on AR in retail has two primary streams. The first stream of literature sheds light on the effects of AR use and delves into the underlying mechanisms. The second stream of literature gains insight into the impacts of specific AR characteristics and reveal how these impacts take place. First, AR use, that is, the inclusion of AR-empowered product presentation and interaction capabilities in retail applications has positive effects on consumer responses to the AR technology/AR retail application (i.e., the web-based AR application and mobile AR app). Specifically, AR use can stimulate favorable consumer attitude toward (Plotkina and Saurel, 2019; Yim and Park, 2019; Smink et al., 2020; Daassi and Debbabi, 2021), increase their satisfaction with (Poushneh and Vasquez-Parraga, 2017), adoption/use intention of (Yim and Park, 2019; Bonnin, 2020), reuse/continued use intention of (Daassi and Debbabi, 2021), and recommendation intention (Smink et al., 2020) of the AR technology/AR retail application. These benefits are achieved through the utilitarian value and hedonic value (Poushneh and Vasquez-Parraga, 2017; Plotkina and Saurel, 2019; Yim and Park, 2019; Bonnin, 2020) that consumers experienced while using the AR retail application.

Second, extant literature examines the impacts of specific AR characteristics such as interactivity, augmentation, informativeness, vividness, novelty, and aesthetics on consumers’ attitudes toward (Pantano et al., 2017; Rese et al., 2017; Yim et al., 2017; Park and Yoo, 2020; Qin et al., 2021b), satisfaction with (Chiu et al., 2021), adoption/use intention of (Pantano et al., 2017; Rese et al., 2017; Park and Yoo, 2020; Qin et al., 2021b), reuse/continued use intention of (Javornik, 2016; Pantano et al., 2017; Chiu et al., 2021; Hsu et al., 2021; Kowalczuk et al., 2021; Nikhashemi et al., 2021), and recommendation intention of (Javornik, 2016; Hilken et al., 2017; Pantano et al., 2017; Park and Yoo, 2020) the AR technology/AR retail application.

Furthermore, compared with the studies focused on the effects of AR use, research on the impacts of AR characteristics delves deeper into the underlying mechanisms of how AR characteristics influence consumers’ responses to the AR technology/AR retail application. Except for the evaluation of the utilitarian value and hedonic value (Hilken et al., 2017; Pantano et al., 2017; Rese et al., 2017; Yim et al., 2017; Hsu et al., 2021; Nikhashemi et al., 2021; Qin et al., 2021b), this stream of literature also proposes and validates a variety of psychological mechanisms, such as affective responses and cognitive responses (Kowalczuk et al., 2021), flow (Javornik, 2016), inspiration (Rauschnabel et al., 2019; Nikhashemi et al., 2021), and mental image (Park and Yoo, 2020).

#### Product-Related Outcome Variables

As shown in Table 4, product-related outcome variables investigated in the literature on AR in retail include consumers’ product attitude, product purchase intention, willingness to pay a price premium, and WOM intention. The majority of the journal articles use consumers’ product purchase intention as the outcome variable. Some articles also examined consumers’ responses to the AR technology/AR retail application at the same time. Consumers’ product attitude refers to their feelings about a product (van Esch et al., 2019; Fan et al., 2020). Consumers’ product purchase intention describes their willingness to purchase the product they experience in the AR retail application (Javornik, 2016; Plotkina and Saurel, 2019; Smink et al., 2019). Consumers’ willingness to pay a price premium refers to their intention to pay a higher price for a product (Nikhashemi et al., 2021). Consumers’ WOM intention refers to their willingness to say positive things about the product to friends, relatives, and other people (Hilken et al., 2017).

Similar to the studies on the impacts of AR on technology-related outcomes, literature on this theme also has two streams. The first stream of literature sheds light on the effects of AR use. The second stream of literature gains insight into the impacts of AR characteristics. First, AR experience/use stimulates the consumer purchase intention by increasing cognitive control (Whang et al., 2021), eliciting higher self-brand connection (Baek et al., 2018), and strengthening the utilitarian value and hedonic value perception (Plotkina and Saurel, 2019; Smink et al., 2019). Furthermore, the literature also reveals the boundary conditions of how AR experience/use impacts their purchase intention. For instance, Whang et al. (2021) show that peer opinions moderate the impacts of AR experience on the consumer cognitive control and purchase intention.

Second, AR characteristics such as interactivity (Hilken et al., 2017; Yim et al., 2017; Kowalczuk et al., 2021; Nikhashemi et al., 2021), vividness (Hilken et al., 2017; Yim et al., 2017; Nikhashemi et al., 2021), augmentation (Javornik, 2016; Fan et al., 2020; Poushneh, 2021), informativeness (Kowalczuk et al., 2021), novelty (Hilken et al., 2017; Nikhashemi et al., 2021), quality (Kowalczuk et al., 2021; Nikhashemi et al., 2021), reality congruence (Kowalczuk et al., 2021), anthropomorphism (van Esch et al., 2019), and sensory control modality (Heller et al., 2019b) affect product-related outcomes. The majority of the studies focus on the consumer product purchase intention. Some studies also shed light on the impacts of AR characteristics on consumers’ product attitudes (van Esch et al., 2019; Fan et al., 2020), willingness to pay a price premium (Nikhashemi et al., 2021), and WOM intention (Hilken et al., 2017). These studies explain the impacts using consumers’ experience of the utilitarian benefits and hedonic benefits (Hilken et al., 2017; Poushneh and Vasquez-Parraga, 2017; Yim et al., 2017; Nikhashemi et al., 2021), consumers’ affective responses and cognitive responses (Javornik, 2016; Kowalczuk et al., 2021), sense of presence (Hilken et al., 2017), sense of immersion (Yim et al., 2017), mental imagery (Heller et al., 2019b), inspiration (Nikhashemi et al., 2021), and flow (Javornik, 2016).

#### Brand-Related Outcome Variables

Compared with the AR marketing literature investigating technology-related and product-related outcome variables, the studies examining brand-related outcome variables are both new and limited in quantity. As presented in Table 4, the brand-related outcome variables include consumers’ brand attitude, perceived brand personality, and brand purchase intention. Among them, consumers’ brand attitude is the most popular one. Moreover, the brand-related outcome variables are usually investigated with the product-related outcome variables (e.g., product purchase intention) and technology-related outcomes (e.g., reuse intention).

Consumers’ brand attitude refers to their feelings about a brand (Rauschnabel et al., 2019; Smink et al., 2019, 2020; van Esch et al., 2019). Consumers’ perceived brand personality describes their systematic and enduring perception of a set of human traits that serve as the foundation of brand relational consequences and brand equity (Plotkina et al., 2021). Consumers’ brand purchase intention refers to their willingness to buy the products of a specific brand (Smink et al., 2020).

Similar to AR marketing literature on product-related and technology-related outcome variables, the journal articles that investigate the effects of AR on brand attitude can be categorized into two groups. First, AR use (i.e., online product presentation with AR) enhances consumers’ brand attitude by eliciting their perception of spatial presence, personalization, and utilitarian and hedonic benefits (Smink et al., 2019, 2020). However, AR use can also be harmful to consumers’ brand attitudes because it may elicit the perception of intrusiveness (Smink et al., 2019).

Second, AR characteristics such as augmentation and anthropomorphism influence consumers’ brand attitudes. In particular, augmentation drives changes in consumers’ brand attitudes through inspiration (Rauschnabel et al., 2019). Anthropomorphism (i.e., endowing AR with human characteristics) influences consumers’ attitudes toward the brand by boosting confidence, increasing the perceived transaction convenience and innovativeness, and decreasing the perceptions of barriers to AR use (van Esch et al., 2019). Furthermore, AR types such as goal and location affect consumers’ perceived brand personality. The impact is mediated by consumers’ perceived AR app experience and attitudes toward the AR app and moderated by consumer characteristics such as IT innovativeness and shopping orientation (Plotkina et al., 2021).

### Tourism

Tourism is an emerging application area of AR marketing. Different from retail in which increasing product sales is the central point, the primary concern for tourism is enhancing visitors’ experience. AR is valuable for the tourism industry in multiple ways, such as economic, experiential, social, epistemic, cultural and historical, and educational (tom Dieck and Jung, 2017). The application of AR in tourism transforms tourists’ experience by providing more interactive, enjoyable, personalized, and context-aware tourism experiences, which further increases tourists’ satisfaction and expands target markets (Jung et al., 2015; tom Dieck and Jung, 2017; Jiang et al., 2019). Therefore, more and more business entities in tourism, such as tourism destinations (Lacka, 2020; Huang and Liu, 2021), heritage tourism sites (tom Dieck and Jung, 2018; Tsai et al., 2020), museums (He et al., 2018), art galleries (tom Dieck et al., 2016, 2018b), protected areas (Jiang et al., 2019), science festivals (tom Dieck et al., 2018a), theme parks (Jung et al., 2015), and restaurants (Heller et al., 2019a; Batat, 2021), adopt AR applications in offline environments and online settings.

The first journal article on AR in tourism was published in 2015, which is just one year after the publication of the first journal article on AR in retail. The number of journal articles exploring AR in tourism keeps increasing over the past few years. Prior studies on AR in tourism examined the influence of AR on various technology-related and tourist destination-related outcomes. Some journal articles also delved into the underlying psychological and behavioral mechanisms. Besides, several journal articles gained insight into the broader themes, such as the perceived value of AR for the tourism industry (tom Dieck and Jung, 2017; Cranmer et al., 2020) and the AR business models in the tourism industry (Cranmer et al., 2020).

#### Technology-Related Outcome Variables

The technology-related outcome variables examined in the literature on AR in tourism are similar to those investigated in the literature on AR in retail. As presented in Table 4, the outcome variables include tourists’ attitude toward, adoption of, satisfaction with, and recommendation intention of the AR technology/AR tourism application. Tourists’ attitude toward the AR technology/AR tourism application refers to their feelings about the AR technology/AR tourism application (Wu et al., 2013; Jung et al., 2018; Paulo et al., 2018; tom Dieck and Jung, 2018; Shin and Jeong, 2021). Tourists’ adoption of the AR technology/AR tourism application describes their willingness to use the AR technology/AR tourism application (tom Dieck and Jung, 2018). Tourists’ satisfaction with the AR technology/AR tourism application refers to their overall feelings while interacting with it constantly within a period (Jung et al., 2015). Tourists’ recommendation intention for the AR technology/AR tourism application describes their desire to publicly or privately share the information about it (Jung et al., 2015).

First, scholars have proposed improved models of the well-recognized models, such as the Technology Acceptance Model (TAM), Unified Theory of Acceptance and Usage of Technology (UTAUT2), and Task Technology Fit (TTF). These models can better explain the determinants of the adoption of AR in tourism by incorporating new antecedents or combining existing models (Wu et al., 2013; Jung et al., 2018; Paulo et al., 2018; tom Dieck and Jung, 2018). Particularly, tourists’ perceptions of usefulness and ease of use of AR technology have significant positive impacts on their attitude toward AR technology. Moreover, tourists’ motivations to adopt AR, such as hedonic motivation, utilitarian motivation, and self-presentation motivation, have significant positive effects on their attitudes toward AR tourism applications (Shin and Jeong, 2021). In addition, tourists’ attitudes toward (Shin and Jeong, 2021; Zhuang et al., 2021) and subjective norms of (Zhuang et al., 2021) AR technology positively impact their intention to use it.

Second, the three quality dimensions of the AR tourism application (i.e., content quality, personalized service quality, and system quality) affect tourists’ satisfaction with and recommendation intention of it. The effect of the quality dimensions on tourists’ intention to recommend the AR tourism application is mediated by their satisfaction with it. Furthermore, this effect is more prominent for the tourists with high innovativeness than for those with low innovativeness (Jung et al., 2015).

#### Tourist Destination-Related Outcome Variables

Tourist destination-related outcome variables include tourists’ knowledge acquisition of, visit intention of, satisfaction with, memory of, and WOM generation for tourist destinations, as well as tourists’ choice of products and willingness to pay a price premium in tourist destinations. Specifically, AR enriches tourists’ sensory, affective, behavioral, social, and intellectual experiences (Heller et al., 2019a). The enriched experiences lead to tourists’ better knowledge acquisition of tourist destinations, increased intention to visit tourism destinations, improved satisfaction with and memory of the tourist destination, choice of higher value products, and increased willingness to pay a price premium. In conclusion, the application of AR in tourism increases the overall well-being of tourists (Batat, 2021).

Tourists’ knowledge acquisition of a tourist destination refers to their learning of new, interesting, or necessary things about it (tom Dieck et al., 2018b; Lacka, 2020). Tourists’ intention to visit a tourist destination describes their desire to visit it (Chung et al., 2018; He et al., 2018; Lacka, 2020). Tourists’ satisfaction with a tourist destination refers to how much they enjoy visiting it using the AR tourist application (tom Dieck et al., 2018a). Tourists’ memory of a tourist destination describes what stays in their minds after visiting it using AR tourist applications (tom Dieck et al., 2018a). Tourists’ choice of higher value products refers to their decision to buy products of higher prices (Heller et al., 2019a). Tourists’ willingness to pay a price premium describes their desire to pay a higher price that exceeds the benchmark price (Huang, 2021).

The studies delving into the underlying mechanisms of the effects reveal that they are achieved by creating an immersive experience, stimulating tourist engagement, and increasing processing fluency (Heller et al., 2019a; Tsai et al., 2020). Moreover, the effects are heterogeneous across tourists with different visual processing styles and sensation-seeking tendencies, and products with different contextuality (Heller et al., 2019a). More nuanced investigations into the impacts of specific AR characteristics provides a deeper understanding of how AR affects tourists. For instance, the two AR empowerment features, such as environmental embedding and simulated physical control, foster immersion and increase the willingness to pay more by generating a restorative experience (Huang, 2021). Moreover, the three dimensions of technology embodiment (i.e., ownership, location, and agency) affect tourists’ enjoyment and enhance their experience (Tussyadiah et al., 2018). The three key features of humanizing experiences in the AR tourism application (i.e., anthropomorphism, self-representation, and intimacy) lead to a more prominent effect on the brand love of tourism destinations (Huang and Liu, 2021). In addition, both the information types (i.e., dynamic verbal vs. visual cues) and the augmenting immersive scenes (i.e., high vs. low virtual presence) influence tourists’ purchase intentions and willingness to pay more. Specifically, dynamic verbal cues lead to a higher level of willingness to pay more than dynamic visual cues. The effect is more prominent in high virtual presence environments (He et al., 2018).

### Advertising

Advertising is the latest and fast-growing application area of AR marketing. The application of AR in advertising is mobile apps based (Yang et al., 2020; Sung, 2021) or in the form of online AR advertisement videos (Feng and Xie, 2018). Compared with traditional print advertising, radio advertising, and TV broadcast advertising, AR advertising is more informative, novel, entertaining, and complex (Feng and Xie, 2018; Yang et al., 2020). AR-enabled immersive, interactive, and personalized experience elicits positive consumer responses and helps advertising campaigns stand out (Sung, 2021).

A variety of advertisement characteristics affect the consumers’ affective, cognitive, and behavioral response to AR advertisements. The characteristics of AR advertisements include AR advertisement type (e.g., quick response hypermedia and app response hypermedia) (Uribe et al., 2021), AR interaction type (e.g., instrumental and hedonic) (Tsai et al., 2020), and advertisement context (e.g., realistic and imaginative) (Tsai et al., 2020). In addition, product type (i.e., think and feel) (Tsai et al., 2020) and consumer personality traits (i.e., extraversion, openness, agreeableness, conscientiousness, and neuroticism) (Srivastava et al., 2021; Uribe et al., 2021) also have influence on the consumers’ responses to AR advertisements. Specifically, AR advertisements enhance consumer physiological responses (Pozharliev et al., 2021), boost their engagement (Sung, 2021), and facilitate social experience sharing among consumers (Sung, 2021). These desirable effects further stimulate positive attitudes toward AR advertisements (Yang et al., 2020), increase the efficacy of advertising campaigns (Feng and Xie, 2018), strengthen consumer-brand connections (Pozharliev et al., 2021), increase the brand liking (Tsai et al., 2020), and stimulate product purchase intentions (Pozharliev et al., 2021; Sung, 2021).

#### Advertisement-Related Outcome Variables

Advertisement-related outcome variables examined in AR advertising literature is consumers’ attitudes toward AR advertisements. It refers to consumers’ feelings toward AR advertisements (Feng and Xie, 2018; Yang et al., 2020; Uribe et al., 2021). Overall, AR advertising has many advantages over traditional ones. Specifically, AR advertising leads to positive attitudes toward the advertisements. This effect is mediated by consumers’ perceived enjoyment and informativeness (Uribe et al., 2021). The content characteristics of AR advertisements, such as informativeness, novelty, entertainment, and complexity, affect consumers’ attitudes toward AR advertisements. Moreover, irritation, value, and believability of AR advertisements serially mediate the effects of the content characteristics of AR advertisements and consumers’ attitudes toward them (Feng and Xie, 2018). Compared with traditional advertisements without AR, advertisements with AR can increase consumers’ curiosity about the advertisements, which in turn attract their visual attention toward the advertisements and bolster their attitudes toward the advertisements (Yang et al., 2020). Besides, AR interaction type, advertisement context, and product type affect the perceived informativeness of AR ads. Telepresence mediates the effects (Tsai et al., 2020).

#### Brand-Related Outcome Variables

Brand-related outcome variables examined in the literature on AR in advertising include consumers’ brand attitude and brand liking. Brand attitude and brand liking are consumers’ feelings about a brand (Phua and Kim, 2018; Tsai et al., 2020; Uribe et al., 2021). AR advertisements have a positive impact on consumers’ attitudes toward the brand. Consumers’ perception of the advertisements’ entertainment value partially mediates the effect (Uribe et al., 2021). Moreover, self-brand congruity, self-referencing, and perceived humor significantly influence consumers’ post-use brand attitude toward the advertised brand (Phua and Kim, 2018). AR interaction type (i.e., instrumental vs. hedonic), advertisement context (i.e., realistic vs. imaginative), and product type (think vs. feel) impose significant impacts on brand liking. Telepresence plays the role of mediator in the relationship (Tsai et al., 2020).

#### Product-Related Outcome Variables

The product-related outcome variable examined in the literature on AR in advertising is consumers’ product purchase intention or willingness to pay. Consumers’ product purchase intention or willingness to pay refers to their desire to buy the advertised product (Phua and Kim, 2018; Pozharliev et al., 2021; Uribe et al., 2021). Compared with traditional advertising, AR advertising improves consumers’ attitudes toward advertisements, enhances their emotional responses (i.e., physiological arousal), and leads to higher product purchase intention or willingness to pay (Pozharliev et al., 2021; Uribe et al., 2021). The positive effect of AR advertising on consumers’ product purchase intention is partly mediated by their entertainment value perception of advertisements (Uribe et al., 2021) or fully mediated by their emotional responses (i.e., physiological arousal) (Pozharliev et al., 2021). Furthermore, self-brand congruity, self-referencing, and perceived humor affect consumers’ product purchase intention. Self-brand congruity interacted with the other two factors to influence brand attitude, while the three factors interacted in pairs to affect consumers’ product purchase intention (Phua and Kim, 2018).

## Contributions and Conclusion

This study makes two important contributions to research in AR marketing. First, we delve into the factors specific to AR marketing research. In addition to the shared aspects such as publication year, publication journal, research design, and research method, we shed light on the factors specific to AR marketing such as application area, application context, AR type, and theoretical lenses. Our analyses show that retail, tourism, and advertising are the major application area of AR marketing research. Specifically, retail is the earliest and most popular application area, advertising is the newest and least investigated application area. Next, most prior studies investigated AR applications in online settings, but only a small portion of the literation examined AR applications in on-site scenarios. Third, mobile AR applications and web-based AR applications are the most prevalent AR type in the three application areas. On-site AR applications, wearable AR applications, and somatosensory device-based AR applications have received little scholarly attention in some of the application areas. Finally, TAM, S-O-R Framework, Self-Referencing Theory, UGT, Equity Theory, Flow Theory, TRA, and UTAUT are the most prevalent theoretical lenses in AR marketing research. These findings offer more comprehensive and integrated perspectives to understand the state-of-the-art of AR marketing research.

Second, we identify the focal themes in the three application areas to illustrate the current status of scholarly works. We obtain the focal themes by the outcome variables used in the empirical studies. The outcome variables describe the effects of AR use in general and specific AR characteristics on the AR technology/AR application, products, brands, tourist destinations, and advertisement campaigns. Our analyses show that technology-related variables, product-related variables, and brand-related variables are the shared outcome variables examined in the literature of more than one application area. Tourist destination-related and advertisement-related outcome variables are studied in the literature on a single application area. Specifically, technology-related outcome variables include consumers’/tourists’ attitudes toward, satisfaction with, adoption/use intention of, continued use/reuse intention of, and recommendation intention of the AR technology/AR applications. Product-related variables include consumers’ product attitudes, product purchase intention, willingness to pay a price premium, and WOM intention. Brand-related outcome variables include consumers’ brand attitudes, perceived brand personality, brand liking, and brand purchase intention. Tourist destination-related variables include tourists’ knowledge acquisition of, intention to visit, satisfaction with, the memory of, and WOM generation for tourist destinations. The advertisement-related outcome variable is consumers’ attitudes toward advertisements. These findings provide a clear guideline to grasp the main streams of the AR marketing literature.

## Future Research Agenda

A systematic literature review integrates research papers in a comprehensive, structured, and analytical way. Therefore, it can identify the gaps in extant literature (Paul and Criado, 2020) and highlight the understudied areas that need further attention (Snyder, 2019). We discuss the important but uncovered topics that flow directly from our literature analysis. Then, we put forward the topics that the authors value but have not been investigated in detail by the extant literature.

### The Effects of Augmented Reality on More Outcome Variables and Mediating Variables

As the methods used in most literature are the survey and lab experiment, the outcome variables examined in the literature are self-reported ones collected through scales. With the proliferation of AR applications, the availability of more data collection methods and data analysis techniques will increase significantly. Thus, future research can investigate additional outcome variables and mediating variables obtained from the AR application systems in natural settings (Pantano et al., 2017; Smink et al., 2020; Castillo and Bigne, 2021; Javornik et al., 2021; Poushneh, 2021; Qin et al., 2021a; Tan et al., 2022) or measured via consumer neuroscience methods (Jung et al., 2021; Pozharliev et al., 2021). For instance, except for the examination of the effects of AR on product purchase intention, WOM intention, recommendation intention, and personal data disclosure intention, future research can shed light on the effects of AR on actual purchase behavior, WOM behavior, recommendation behavior, personal data disclosure, post-purchase product satisfaction, customer retention, and product return rate (Smink et al., 2019; Kowalczuk et al., 2021; Qin et al., 2021a). In this vein, we can gain broader insight into more nuanced consumer response and behavior; and obtain a deeper understanding of the affective, cognitive, and social processes underlying the effects of AR on consumer response and behavior.

Furthermore, most existing AR research examined consumers’ immediate experiences and behavioral intentions toward AR. Future research can explore the persistent impacts of AR adoption and design features on consumers’ motivation, experiences, responses, and behavior in various contexts using longitudinal approaches (He et al., 2018; Carrozzi et al., 2019; Huang et al., 2019; Zhang et al., 2019; Barhorst et al., 2021; Batat, 2021; Hsu et al., 2021; Javornik et al., 2021; Uribe et al., 2021). Overall, a more comprehensive understanding of what AR brings to various fields of marketing will enable us to better incorporate this novel and potentially disruptive technology in the service frontline design and operation.

### The Heterogenous Effects of AR

The effects of AR are heterogeneous across consumers with different characteristics, products/services in different categories, and scenarios in different contexts. Although existing literature examines the heterogeneous effects of AR across some general characteristics of consumers, products/services, and contexts, future studies can delve deeper into more nuanced effects of AR regarding consumers, products, services, and contexts with different AR relevant characteristics.

With the fast-growing the application of AR in marketing, consumers using these applications, and products/services offered through these application, future research can delve deeper into the heterogenous effects of AR on consumers’ responses and behavior across consumer characteristics such as gender (tom Dieck et al., 2018a; Smink et al., 2019; Batat, 2021; Chen et al., 2021; Daassi and Debbabi, 2021; Javornik et al., 2021; Yuan et al., 2021), age groups (e.g., children, middle-aged people, and elder) (Jung et al., 2015; Pantano et al., 2017; Plotkina and Saurel, 2019; Smink et al., 2019; Batat, 2021; Chiu et al., 2021; Daassi and Debbabi, 2021; Kowalczuk et al., 2021; Plotkina et al., 2021; Qin et al., 2021b; Yuan et al., 2021), educational level (He et al., 2018; Yuan et al., 2021), occupation (Song et al., 2019), culture background (Jung et al., 2015, 2021; Rese et al., 2017; Rauschnabel, 2018; Jiang et al., 2019; Plotkina and Saurel, 2019; Jessen et al., 2020; Chen et al., 2021; Chiu et al., 2021; Javornik et al., 2021; Lim et al., 2021; Plotkina et al., 2021; Qin et al., 2021a; Yuan et al., 2021), personality traits (Tussyadiah et al., 2018; Park and Stangl, 2020; Uribe et al., 2021), cognitive style (Fan et al., 2020), processing style (Heller et al., 2019b), innovativeness (Huang and Liao, 2015; Huang, 2019; Smink et al., 2019; Yim and Park, 2019; Daassi and Debbabi, 2021), expertise regarding the products/services (e.g., novice vs. experienced) (He et al., 2018; Jung et al., 2021), need for touch (e.g., high vs. low) (Hilken et al., 2017; Huang, 2019; Plotkina and Saurel, 2019), need for vision (e.g., high vs. low) (Huang and Liao, 2017), technology awareness and enthusiasm (Yang et al., 2020), familiarity with AR technology (Park and Yoo, 2020), and privacy sensitivity (Smink et al., 2020; Daassi and Debbabi, 2021).

Future research can also examine the heterogeneous effects of AR on consumers’ responses and behavior regarding different product or service characteristics such as product type (e.g., hedonic vs. functional) (Chen et al., 2021; Pozharliev et al., 2021), product category (Pantano et al., 2017; Song et al., 2019; Park and Yoo, 2020; Barhorst et al., 2021; Castillo and Bigne, 2021; Daassi and Debbabi, 2021; Hsu et al., 2021; Kowalczuk et al., 2021; Plotkina et al., 2021; Whang et al., 2021), product size (small sized vs. large sized) (Yim et al., 2017), product novelty (highly specialized vs. newly developed products) (Hilken et al., 2017), level of body involvement (e.g., high, moderate, and low) (Yim and Park, 2019; Daassi and Debbabi, 2021), and brand awareness (Song et al., 2019).

Moreover, future research can investigate the effects of AR on consumers’ responses and behavior under the contexts with different characteristics such as customer experience stages (e.g., pre-adoption vs. post-adoption, pre-purchase vs. post-purchase, pre-trip vs. post-trip) (Park and Stangl, 2020), noise levels in the ambient environment (e.g., high-noise vs. low-noise environment) (Yang et al., 2020), choice situations (Kowalczuk et al., 2021), and privacy of the environment (i.e., public vs. private) (Javornik, 2016; Carrozzi et al., 2019; Castillo and Bigne, 2021).

### The Effects of Specific Augmented Reality Design Elements and Features

As both the industry practice and academic investigation of AR marketing are still in the infant stage, most existing studies focus on the effects of AR use or AR characteristics. In this regard, deeper investigations of the impacts of sophisticated AR design features in marketing applications on the outcome variables that describe consumers’ experiences of and responses to the AR technology/AR application, products/services, brands, tourist destinations, and advertisements are needed (McLean and Wilson, 2019).

Particularly, to provide enriched information and offer rapid responses to consumers, further research needs to focus on the AR design elements and features that can increase the realisticness, authenticity, vividness, novelty, interactivity, and efficiency (Huang and Liu, 2014; Pantano et al., 2017; Bonnin, 2020; Jessen et al., 2020; Barhorst et al., 2021). For instance, current AR marketing studies build upon AR applications that augment consumers’ visual and auditory perceptions of products and services. With the emergence of the AR technology that can enrich more sensory experiences such as tactile, gustatory, and olfactory, new AR applications using it can provide multi-sensory feedback (Heller et al., 2019b). Thus, scholars can seek to investigate the effects of AR applications incorporating multi-sensory augmentation and feedback capabilities (Huang and Liao, 2017; Heller et al., 2019a,b; Sung, 2021).

In conclusion, academic research can help better understand how AR design elements and features will affect consumers’ motivations, experiences, responses, and behavior regarding products/services, brands, and product/service providers. Also, product/service providers can figure out ways of improving AR applications and better satisfying the needs of consumers. Finally, the stakeholders can reach a win–win situation in which both consumers will derive high experiences value and product/service providers can achieve high-revenue profit (Huang and Liu, 2014).

### The Dark Side of Augmented Reality Application in Marketing

Most extant AR marketing literature focus on the bright side of AR use and the positive effects of AR characteristics. However, little studies discuss the dark side of AR application in marketing. To provide enriched personalized services (e.g., consumer movement detection and synchronized and accurate response provision), AR applications need to collect, process, store, and transmit a variety of consumer data such as the face, body, and personal space (Huang et al., 2019; Smink et al., 2019). Thus, potential ethical issues regarding privacy, surveillance, and security risk need more investigations in the future (Rauschnabel, 2018; Carrozzi et al., 2019; Smink et al., 2019; Chang, 2021; Huang and Liu, 2021; Javornik et al., 2021; Lim et al., 2021). For instance, consumers’ privacy concerns may act as a boundary condition and strengthen/weaken the effects of AR use or AR characteristics on their motivations, experiences, response, and behaviors (Lim et al., 2021). Moreover, an underwhelming AR experience will harm consumers’ product/service perception and damage brand equity (Rauschnabel et al., 2019). Another potential outcome of the application of AR is vicarious consumption. Specifically, instead of interacting with the physical elements of a brand, consumers may only build connections with the brand in a computer-mediated environment (Rauschnabel et al., 2019). Therefore, future research needs to hold a more balanced and critical perspective to examine when AR applications will backfire and lead to undesired spill-over effects (Rauschnabel et al., 2019).

## Limitations

Although this research has many meaningful contributions, we acknowledge that this research still has several limitations. These limitations provide opportunities for further investigation. To begin with, the journal articles included in this research are extracted and selected according to our criteria. Thus, we may miss some valuable materials. For instance, we use the WOS core collections as the data extraction source to ensure the high quality of the literature analyzed in this research. Future research can extract literature from more databases such as Scopus, Elsevier, Emerald, Wiley, and Google Scholar to incorporate information from conference proceedings, research reports, working papers, theses and dissertations, books, magazines, white papers, and industry reports. Including more sources and casting the net wider help to gain additional insights. Also, as AR marketing research is still in the infant stage, we use descriptive analysis and thematic analysis in the systematic review. Moreover, the literature analysis is based on the authors’ expertise and understanding. Therefore, the results of this research may have limited generalizability. With the increase in the number of publications on AR marketing, future research can gain more insights into the increased literature by using analytic techniques such as meta-analysis, bibliometric analysis, and text mining.

## Author Contributions

ZD and JL designed the research, collected the data, and conducted the data analysis. ZD, JL, and TW contributed to the drafting and revision of the manuscript. All authors approved the submitted manuscript.

## Conflict of Interest

The authors declare that the research was conducted in the absence of any commercial or financial relationships that could be construed as a potential conflict of interest.

## Publisher’s Note

All claims expressed in this article are solely those of the authors and do not necessarily represent those of their affiliated organizations, or those of the publisher, the editors and the reviewers. Any product that may be evaluated in this article, or claim that may be made by its manufacturer, is not guaranteed or endorsed by the publisher.
